# The Shared and Distinct Mechanisms Underlying Fear of Evaluation in Social Anxiety: The Roles of Negative and Positive Evaluation

**DOI:** 10.1155/da/9559056

**Published:** 2025-04-21

**Authors:** Wei Gao, Yanping Li, JiaJin Yuan, Qinghua He

**Affiliations:** ^1^Institute of Brain and Psychological Science, Sichuan Normal University, Chengdu, Sichuan, China; ^2^Sichuan Key Laboratory of Psychology and Behavior of Discipline Inspection and Supervision, Sichuan Normal University, Chengdu, China; ^3^Faculty of Psychology, Southwest University, Chongqing, China

**Keywords:** emotion, fear, motivation, social anxiety, social pain

## Abstract

Social anxiety disorder (SAD) is associated with persistent fear of negative evaluation (FNE) and fear of positive evaluation (FPE), which play critical roles in the development and maintenance of anxiety symptoms. However, it remains unclear how FNE and FPE contribute to the common and different symptoms of social anxiety. In this review, we tried to elucidate the shared and distinct mechanisms underlying fear of evaluation and clarify the impact of FNE and FPE on social anxiety by integrating the theories, external expressions, and internal mechanisms. First, FNE and FPE share evolutionary functions but have distinct motivations for maintaining social role stability. Second, FNE and FPE share similar emotions and avoidance behaviors but contribute to distinct comorbid symptoms in SAD, including eating disorders and alcohol abuse. Third, FNE and FPE share emotional and social pain circuits but have different dysfunctions in the prefrontal, cingulate, and reward brain regions, which are associated with rejection sensitivity and anhedonia features. Overall, this review sheds light on the cognitive and neural mechanisms of SAD based on fear of evaluation, highlighting both the shared and distinctive aspects of FNE and FPE. These insights have important implications for the development of effective interventions for social anxiety.

## 1. Introduction

Social anxiety is a mental state characterized by fear or anxiety in social or public situations, leading individuals to experience excessive fear and avoidance behaviors in social situations [1]. The early cognitive-behavioral model of social anxiety suggests that fear of evaluation is a core cognitive bias that contributes to the emergence of social anxiety and is also considered an important feature in the development and maintenance of its symptomatology [2] and even develops into social anxiety disorder (SAD) [3, 4]. Therefore, it is important to clarify the effect and mechanism of positive and negative evaluation on social anxiety for improving and treating SAD.

Fear of negative evaluation (FNE) is the worry when facing negative evaluations (e.g., fear of criticism, embarrassment, or rejection), overestimating the likelihood of negative evaluations and consequences, leading to maladaptive emotional, cognitive, and behavioral responses [5]. Studies have found a close association between FNE and social anxiety in adolescent and adult samples [6], with FNE being considered a core criterion for diagnosing SAD. However, some clinical features of social anxiety cannot be fully explained by FNE, such as positivity impairments in which individuals fail to derive emotional benefits from positive feedback [7]. Follow-up studies found that SAD individuals generally experience fear of evaluations without regard to valence and develop fear from not only negative evaluations but also positive evaluations [8]. Fear of positive evaluation (FPE) refers to the worry when receiving positive evaluations from others [9]. For example, a child who gets good grades but is afraid of being praised by their parents. It is probably because the child is afraid of failing to meet expectations (e.g., failing a subsequent test) and thus disappointing the person who evaluated them positively [10]. Therefore, earlier researchers believed that FPE ultimately stems from the FNE and is a delayed expression of FNE [11].

However, the research evidence and reality do not support this view. First, both cross-sectional and longitudinal studies indicate clear distinctions between FNE and FPE [12, 13]. These distinctions will be further elaborated on in subsequent sections. Second, an alternative explanation is also plausible that FPE is a strategy to avoid standing out rather than a delayed expression of FNE, because positive evaluations can attract group attention and lead to competitive pressures [14]. For instance, in reality, there are cases where highly competent employees fear public praise because they are concerned about the potential conflict consequences (such as competition among colleagues). Third, several studies emphasized the necessity of developing distinctive diagnostic criteria and treatment for FPE because some therapies that can help improve FNE (e.g., positive feedback) may not be suitable for individuals with FPE characteristics [15]. Given the confusion of the available evidence, we would like to give a systematic review by clarifying theoretical explanations, external expressions, and internal mechanisms of FNE and FPE to explain how positive and negative evaluations impact social anxiety.

## 2. Theoretical Explanations of FNE and FPE

### 2.1. The Shared Evolutionary Functions of FNE and FPE

**Figure 1:**
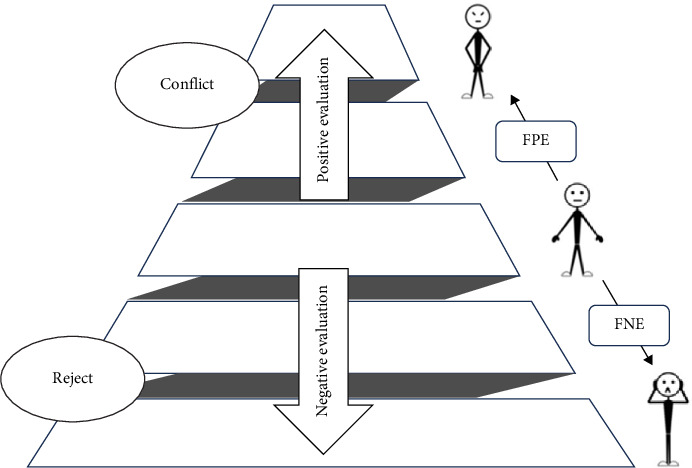
Bivalent fear of evaluation model (BFoEM; [17]).

Evolutionary psychology provides a theoretical explanation for understanding the relationship between FNE and FPE, suggesting that human societies are similar to biological populations in nature, with a hierarchical organization to help maintain social stability and development [16]. Based on this notion, researchers have proposed the bivalent fear of evaluation model (BFoEM) [17] (Figure 1). This model proposes that individuals with social anxiety tend to consider their surrounding environment as hierarchically structured and perceive themselves to be in a relatively low position in society, so they aim for a stable middle position [18]. As social beings, individuals rely on social relationship networks composed of approval and support, while negative evaluation may be a threat to the fundamental need for social proximity in humans [19]. Negative evaluations can lead to a downward shift in an individual's social status, eventually resulting in social exclusion, which represents a typical threat signal to survival in the natural world. Therefore, from an evolutionary view, FNE can help reduce antisocial behavior and increase prosocial behavior, avoiding social exclusion [20].

In contrast, the function of FPE is to protect oneself from conflict and threats by avoiding leaving a “too good” impression, as the implicit upward mobility in positive evaluations attracts group attention, prompting other members to compete and challenge the “rising star” [ 21 ]. It has been shown that the capacity for gratification and interpersonal needs positively correlate with well-being and psychological adjustment across cultures [22]. Thus, in the process of maintaining a “low-profile” intermediate position, FPE and FNE work as two moderating forces that synergize to help individuals find an equilibrium that prevents them from rising or falling in social status. This may be why individuals with social anxiety, regardless of valence, exhibit fear of both negative (criticism, rejection, potential downward shift) and positive (praise, recognition, potential upward shift) evaluations, as they are sensitive to both types of evaluations and perceive them as potential social threats [23, 24].

### 2.2. The Distinct Motivations of FPE and FNE

Positive and negative evaluations are both perceived as threats to individuals with social anxiety. However, there is controversy regarding whether FPE can be considered a delayed expression of FNE. Several researchers proposed that the motivation behind FPE is to avoid future negative evaluations [10]. This explanation suggests that initial positive evaluations raise standards, and if these standards cannot be maintained, negative evaluations will occur. From this perspective, FPE may be a component of FNE, with overlapping structures between the two.

However, based on the BFoEM, there are different motivational directions of FNE and FPE: social exclusion drives FNE to avoid downward social mobility, while the potential consequences of upward mobility (conflict and competition) drive FPE [17]. Other researchers also found that FPE can independently influence a characteristic of social anxiety, and the different motivational directions may be the reason for the structural separation of FPE and FNE [25]. Previous studies conducted factor analyses on scores from FNE and FPE scales separately, they found that the underlying factors of FPE and FNE are highly correlated, but the two-factor structure fits better than the single-factor structure, indicating that FNE and FPE are two separable concepts [26, 27]. Several studies also assessed the structural components of FPE and observed that in addition to the overlapping components with FNE. There are also some unique components related to: (1) lack of approval for positive social outcomes (underestimating and minimizing successful positive interactions) and (2) concerns about social retaliation (competition triggered by positive evaluations). Furthermore, the results of studies in different samples also found that compared with clinical patients, healthy individuals are better able to distinguish between reactions to negative and positive evaluations when facing social evaluations, and the correlation between FNE and FPE is significantly lower in healthy people compared to clinical patients, suggesting function independently with FNE and FPE [28, 29]. Moreover, it has been shown that people with strong group needs may hide or minimize their successes, so individuals in collectivist cultures may have higher FNE to avoid social exclusion [22]. These findings support that there is a close association between FNE and FPE, but the two types of fear still have distinct motivations that contain their unique components.

## 3. External Expressions of FNE and FPE

### 3.1. The Shared Emotions and Behaviors of FNE and FPE

It has been demonstrated that individuals with SAD are primarily characterized by their abnormal attention to and response to social threat stimuli. Previous studies indicated that both FNE and FPE are accompanied by increased avoidance behaviors in social situations, such as more avoidance gestures (avoiding eye contact, speaking softly) when participating in group activities or talking to older colleagues [30]. Moreover, excessive attention to positive and negative evaluations is associated with individuals' lower self-esteem and negative self-judgments [31]. Besides, clinical samples have also reported that both FNE and FPE were associated with maladaptive social feedback and responses, which lead to a significant decrease in the quality of life and personal accomplishments in SAD [3].

In addition, prior researchers explored the relationship between SAD and other psychopathological symptoms and suggested that FNE and FPE may interact on the comorbid characteristics of these diseases, with both being considered vulnerability factors leading to mental disorders [14, 32]. For example, several studies have reported that people with FNE and FPE have a negative processing bias for social information that is not only commonly found in SAD but also in personality disorders and autism [33]. Furthermore, results of social interaction studies also found that both positive and negative evaluation scenarios can lead to one's withdrawal behavior and induce fear reactions in SAD, and these strong negative emotions are consistently found in general anxiety and depression [34, 35]. Research in both collectivist and individualist cultural contexts has shown that individuals with FPE and FNE have a bias to interpret external feedback negatively and are positively correlated with social anxiety [36, 37]. These studies suggested that FNE and FPE individuals have a common negative bias in cognitive processing, resulting in similar emotions and external avoidance behaviors.

### 3.2. The Distinct Symptoms and Contributions of FNE and FPE

Beyond some common features of FNE and FPE, recent evidence suggests that each has its distinctive contribution as well [38]. For example, prior studies indicated that the FNE and FPE scores can only unilaterally predict an individual's unpleasant reaction to negative or positive evaluations, but they cannot predict each other, regardless of whether evaluation materials are presented in text, images, or video [19]. Similarly, the results of the longitudinal study showed that FNE and FPE can make prospective predictions for themselves but no such prospective predictive relationship between each other. The mutual unpredictability supports distinct symptoms in FNE with excessive negative expectations and FPE with reduced compensatory pleasure.

Previous studies also supported that FNE and FPE have distinct contributions by examining the relationship between different mental disorders and fear of evaluation. On the one hand, the close correlation between FNE and high rejection sensitivity resulted in a specific association between FNE and body image anxiety and eating disorders. Rejection sensitivity refers to the degree to which individuals fear, perceive, and behave in excessive ways toward social exclusion [39]. College students with high FNE levels reported severe symptoms of body image concerns and eating problems, and some studies also reported a positive correlation between FPE and body dissatisfaction, but the correlation was not significant after controlling for FNE levels [40]. Individuals with high FNE are more likely to anticipate appearance conditioning as a rejection sensitivity cue for social exclusion and easily perceive negative evaluations of body image from others, finally leading to body image anxiety and eating disorders [41]. On the other hand, compared to FNE, FPE is associated with anhedonia that causes the individual's substance dependence, such as drinking. In general, people with FNE can derive pleasure from positive evaluations to compensate for negative evaluations like normal people. However, anhedonia may prevent individuals with FPE from deriving pleasure from positive evaluations, and they may have to seek other substances to supplement pleasure [42]. For example, some studies have found that individuals high in FPE show stronger hedonic drinking motives and drinking behavior. Because drinking not only blurs social hierarchies but also elevates physical arousal and enhances pleasurable feelings [11, 43]. Other studies also examined the correlation between FNE and drinking by using FNE and FPE as simultaneous predictors, but the results showed that only FPE remained significantly associated with the outcome. It has also been found that after controlling for FNE and gender, FPE is still strongly associated with drinking problems [44]. In addition, a longitudinal study also highlighted the unique contribution of FPE and found that anhedonia mediated the relationship between FPE and depression [45]. Furthermore, the experience of social anxiety varies across cultures, and cross-cultural research on social anxiety suggests that individuals from collectivist countries report greater social anxiety and FNE than individuals from individualist countries. Since individuals from collectivist countries may be less likely to “show off” than individuals from individualist countries, they may have less reason to fear positive evaluations. However, the FPE is significantly higher in individualistic countries than in collectivistic countries [46]. In sum, these findings suggest the distinct symptoms induced by FNE and FPE in mental disorders.

## 4. The Internal Mechanisms in FNE and FPE

### 4.1. The Shared Social Pain Circuits of FNE and FPE

As mentioned earlier, individuals with FNE and FPE prefer to concern themselves with negative social information and perceive negative evaluations as signals of social exclusion, even interpreting positive social feedback negatively. For example, high FNE individuals showed rapid orienting to negative emotional faces in a social evaluation experiment (preparing a speech) [47], while high FPE individuals tend to interpret positive evaluation information in a negative way (ridicule or sarcasm) in social interactions, resulting in negative emotions [48]. These negative cognitive biases and negative emotion generation processes may be attributed to social pain circuits associated with social exclusion, including the cingulate gyrus, amygdala, and insula [49]. Previous studies observed that the dorsal anterior cingulate cortex (dACC) is activated when individuals perceive feedback signals of social rejection and correlated with subjectively reported levels of social pain, whereas levels of dACC activation decrease after being treated with medication used to alleviate social pain [50, 51]. Moreover, research on social anxiety indicated that the dACC plays a role in monitoring and disengaging from socially threatening stimuli and is associated with goal conflict and expectation mismatch, which may be involved in the negative cognitive biases process [52, 53]. Furthermore, recent studies also found that people with social anxiety showed abnormal activities in the dACC in evaluative situations when they were asked to watch social criticism and praising words [54]. These findings suggest that the dACC may contribute to FNE and FPE by getting involved in processing social pain information.

It has been documented that the amygdala and insula are primarily involved in emotion generation processes, especially in responses and internal feelings associated with fear [55], such as excessive somatic reactions and symptoms (headaches, cold sweats, and gastrointestinal distress) [47]. Prior researchers examined people's reactions when confronted with potentially threatening stimuli (e.g., harsh faces, public speeches, verbal criticism) and observed increased bilateral amygdala and insula activations in the social anxiety group compared with the healthy group, and the degree of activation was positively correlated with the severity of anxiety symptoms [56]. Moreover, developmental psychology studies indicated that adolescents with more severe social anxiety symptoms than older adults showed hyperactivation of the bilateral amygdala and insula in social evaluation experiments, which has been linked to “social pain” neural circuits [57]. Besides, several studies found abnormal connectivity in the amygdala and insula with the prefrontal cortex when individuals read negative evaluations, and a recent study also found abnormal activities in the amygdala and insula when individuals watched positive and negative evaluative videos compared to neutral conditions. These findings suggest that the amygdala and insula may support the anxious inner feeling and the excessive fear reaction of FNE and FPE [58]. Therefore, we hypothesize that FNE and FPE in both collectivist and individualist cultural contexts are associated with activating the amygdala and insula. More cross-cultural and imaging studies are needed to demonstrate this in the future.

### 4.2. The Distinct Internal Mechanisms of FNE and FPE

Although FNE and FPE share common mechanisms of fear feelings and avoidance behavior, there are differences in the production processes of FNE and FPE, which are dominated by the enhancement of negative pain and the deprivation of positive pleasure, respectively. FNE individuals have an attentional bias towards negative evaluative information involving social exclusion but not positive evaluative information, which is attributed to the association between FNE and high rejection sensitivity. SAD with higher rejection sensitivity activates social pain-related brain regions, including the anterior insula, anterior cingulate, and ventrolateral prefrontal cortex (VLPFC) [59, 60]. The VLPFC is considered to modulate the perception of evaluation information and painful experiences in healthy people according to the model of cognitive-affective system theory with rejection sensitivity. For instance, prior researchers found participants activated the subgenual ACC, the VLPFC, and the ventral striatum during social exclusion, and painful experience is negatively correlated with activity in the VLPFC and ventral striatum region [61, 62], which suggested the regulatory function of VLPFC and ventral striatum in negative emotions. Moreover, several studies also found that individuals who are criticized often alleviate pain after rejection through self-regulatory processes by relying on functional couplings in the VLPFC and ventral striatum, which have been shown to exert inhibitory effects on the ACC regions [49, 50]. In addition, by contrasting criticism and praise conditions, previous studies also indicated that FNE, but not FPE, was associated with neural response to social evaluation and self-regulation [63]. Therefore, we speculate that amplified negative expectations and uncontrollable fear in SAD with FNE are associated with abnormal activity in the VLPFC and ventral striatum regions, which play moderating roles in social pain circuits.

On the other hand, SAD patients with FPE are unable to derive pleasurable feelings from the praise of others, which rarely happens in individuals with FNE alone. The potential explanation is that FPE individuals, rather than FNE individuals, have a negative interpretation bias and reduce the value of positive evaluative information, resulting in individuals not benefiting from positive social feedback. Previous studies have found anhedonia in people with FPE, involving abnormal prefrontal and reward brain activities, including the orbitofrontal and ventromedial prefrontal cortex (VMPFC), ventral tegmental area (VTA), and ACC regions [64, 65]. Specifically, anhedonia is often observed in psychiatric disorders such as depression and anxiety, which are often associated with lower appraisal and experience of reward value. Processing of reward is complex but is almost certainly mediated by dopaminergic neurons originating from the VTA and extending to the ventral striatum, orbitofrontal cortex (OFC), VMPFC, and ACC to form an integrated midbrain limbic dopamine pathway [66]. Moreover, a series of research evidence showed that individuals have increased functional connectivity between VTA and the right medial PFC and ACC regions, which are correlated to more severe symptoms of anhedonia in SAD [67, 68]. Furthermore, the OFC is intricately involved in the evaluation of value, including the assessment of reward and punishment associated with decision-making processes. This brain region integrates sensory information with emotional and reward-related signals to assign value to various stimuli and outcomes. Dysfunction in the OFC can lead to impairments in value-based decision-making, contributing to difficulties in assessing the potential outcomes of actions and making appropriate choices [69]. This dysfunction may manifest in SAD with FPE, where individuals may exhibit maladaptive behaviors driven by distorted perceptions of value, such as underestimating the reward value of positive social evaluations and reducing the likelihood of positive outcomes. In addition, studies of other mental disorders have also suggested the function of OFC in anhedonia; for example, it was found that reduced gray matter volume in the OFC and caudate nucleus was associated with the experience of pleasure deficit in depressed patients [70]. Moreover, it has been shown that individuals in collectivist cultures have higher FNE than FPE, whereas the opposite is true in individualist cultures. Therefore, we hypothesize that individuals in collectivist cultures activate the VLPFC and ventral striatum due to rejection sensitivity, and individuals in individualist cultures activate the OFC and VTA due to anhedonia. Taken together, it is plausible that dysfunction of the OFC and VTA is involved in FPE, leading to reduced compensatory pleasure. Given the limited evidence for comparative studies of FNE and FPE, it is necessary to investigate the neural mechanisms of FNE and FPE and to verify our findings in the future.

## 5. The Shared and Distinct Impacts on Social Anxiety by FNE and FPE

**Figure 2:**
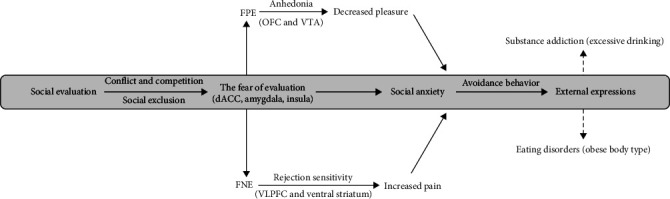
The shared and distinct impacts on social anxiety by FNE and FPE. FNE, fear of negative evaluation; FPE, fear of positive evaluation.

Although the existing research findings are varied, a large number of previous studies have confirmed that FNE and FPE may contribute to the common and different symptoms of social anxiety [30, 71,72–74]. Therefore, based on the shared and distinct mechanisms of FNE and FPE mentioned in the above paragraphs, we tried to organize and integrate the evaluation of fear-related theories, mechanisms, and disorders to elucidate the processes underlying social anxiety induced by FNE and FPE (Figure 2):

(1) The theoretical explanation of BFoEM provides support for understanding the evolutionary function of FNE and FPE, which were regarded as “regulatory forces” that help individuals maintain stability in social hierarchies to avoid upward or downward shifts in their social status. From this perspective, the similarity between FNE and FPE is that both can be regarded as potential social threats, which is the reason why people with social anxiety are afraid of all evaluations regardless of valence. The differences between FNE and FPE are shown in the motivational direction, including prevention of downward social mobility (social exclusion) through FNE and avoidance of the potential consequences of upward mobility (conflict and competition) through FPE, the separation of motivation at the input stage of evaluative information may lead to differences in internal mechanisms.

(2) The shared internal mechanism of FNE and FPE benefits from social pain circuits, including the dACC, amygdala, and insula. These brain regions support similar patterns of cognitive and emotional processes to evaluative information, which contribute to convergent external expressions such as negative cognitive biases and fear-emotional responses. The separated internal mechanisms of FNE and FPE can be attributed to the different associations between FNE and rejection sensitivity, as well as FPE and anhedonia. FNE with rejection sensitivity involved abnormal activities of VLPFC and ventral striatum, resulting in uncontrollable fear and feelings of pain, while FPE with anhedonia involved abnormal activities of OFC and VTA, resulting in reduced compensatory pleasure. Increased pain and decreased pleasure in the generation stage may cause different symptoms of social anxiety and contribute to comorbidity with other disorders.

(3) The similar effect of FNE and FPE results in consistent avoidance behavior and anxiety symptoms, which may be a potential factor in the widespread association of social anxiety with other disorders. In contrast, the different comorbid symptoms associated with social anxiety may reflect the unique effects of FNE versus FPE differences. On the one hand, individuals with FNE are more likely to perceive and anticipate fear from social exclusion cues (such as an obese body type), so they will adjust their eating behavior more frequently, resulting in a unique association between FNE and eating disorders. On the other hand, the anhedonia characteristic of FPE individuals may cause a failure to receive positive feedback compensation from positive evaluations, so they have to attempt to obtain pleasure through substance use, which may be one of the reasons for the unique association between FPE and substance addiction (e.g., excessive drinking). Overall, the current model tries to systematically describe the process of social anxiety arising from negative and positive evaluations, aiming to elucidate the relationship between different types of evaluative fears and social anxiety and revealing the mechanisms and contributions of FNE and FPE to social anxiety. Given the mixed results of previous studies and the fact that there is still insufficient research on FNE and FPE, the reliability and validity of this model need examination in future work.

## 6. Limitations and Future Directions

Collectively, the current systematic reviews extend the bivalent fear of the evaluation model to internal neural mechanisms, which facilitate our understanding of FNE and FPE, as well as have important clinical implications. First, the similarities and differences in neural circuits support the prior notion that FPE and FNE are distinct, yet correlated, trait-based social evaluative fears that uniquely contribute to social anxiety symptomatology. Second, given that individuals with both FNE and FPE experience greater psychological difficulties, the potential neurological evidence would promote including both FNE and FPE in the diagnostic criteria for SAD. Third, although FPE is sensitive to cognitive-behavioral treatments, effect sizes were slightly smaller compared to FNE. It may be helpful to develop specific treatments for social anxiety that focus on FNE or FPE based on their different internal mechanisms. Therefore, we would like to give several directions to be explored and clarified to help researchers understand the unique effect of FNE and FPE on social anxiety in future works.

First, research paradigms on positive and negative evaluation fears should be refined and innovated. Currently, there is no widely accepted research paradigm for evaluating fear. Despite numerous attempts by previous researchers, experimental designs still have many shortcomings. On the one hand, directly subjecting participants to social evaluation through text, images, and videos may not effectively induce fear responses. Therefore, it may be necessary to incorporate objective physiological indicators into experiments, as research has found a positive correlation between FNE and FPE and heart rate changes during anticipated speech tasks [75]. On the other hand, methods based on social feedback involve social comparison, leading to confounding results and difficulties in interpretation [54, 76]. Therefore, researchers suggest that in future studies, participants can first perform a test-like task, followed by an evaluation of their test results in subsequent tasks, to avoid interference from social comparison and improve self-involvement and ecological validity to examine individual responses to different types of evaluation fears. Additionally, based on the evaluation fear process model proposed in this article, future experimental paradigms should consider the following: (1) In the social anxiety input stage, previous studies have used mixed evaluation stimuli, resulting in the inability to induce fear or confusion with social comparison elements. Therefore, it may be necessary to clearly distinguish between types of social evaluations, including subjective evaluations, objective evaluations, standard evaluations, comparative evaluations, and so on, to clarify how individuals encode different social evaluations; (2) In the social anxiety generation stage, individuals will experience evaluation fears in different directions influenced by positive and negative aspects. However, previous studies have not differentiated between populations with different types of evaluation fears; some individuals may fear both types of social evaluations, while others may only fear one type. Therefore, future research should differentiate populations with different types of evaluation fears and compare the processes of fear emotions when facing positive and negative evaluations to examine the similarities and differences between the two, which will help identify the unique internal mechanisms of positive and negative evaluation fears; and (3) In the social anxiety response stage, previous research has mainly focused on the relationship between evaluation fear and social anxiety or other mental disorders, without delving into the impact of evaluation fear on comorbid symptoms. Therefore, future studies should not only measure individuals' social anxiety but also examine other disease symptoms related to social anxiety, which will help researchers clarify the contributions of positive and negative evaluation fears to different comorbid symptoms.

Next, it is necessary to examine the group differences in evaluation fear further. On the one hand, as mentioned earlier, previous research has shown that individuals from collectivist countries report a greater FNE than individuals from individualist countries, while individuals from individualist countries have a significantly higher FPE than collectivist countries [46]. In collectivist cultures, modesty is a widely used strategy to avoid conflict with others. Moreover, individuals in collectivist cultures usually view their success as a collective success and have a greater sense of collective honor, so individuals in collectivist cultures do not develop much FPE [37, 77]. However, success in individualistic cultures is primarily beneficial to the individual rather than the collective, and individuals may face subsequent negative evaluations and social retaliation due to the jealousy of others [77]. On the other hand, although this article emphasizes the conceptual separation of FNE and FPE, as mentioned earlier, FPE may also stem from delayed FNE. For example, cross-cultural studies have found a lower correlation between FNE and FPE in Chinese populations compared to Western populations, which may be explained by cultural differences between East and West [46]. Compared to Western individuals, the humility tradition in Eastern cultures (“the tallest trees are cut down by the wind”) may lead to Eastern individuals experiencing FPE more from avoiding conflicts and competition brought about by positive evaluations, while Western individuals' FPE may stem more from delayed FNE. Therefore, it may be necessary to compare differences in positive and negative evaluation fears between Eastern and Western cultural groups. Additionally, current research on social anxiety in adolescent samples has found gender differences in FNE and FPE (with females exhibiting higher levels of evaluation fear) [27]]. However, further examination of differences between males and females in social evaluation fears needs to be conducted in different samples and experiments. For example, investigating whether the relationship between FNE and eating disorders is higher in female groups, while the relationship between FPE and drinking behaviors is more pronounced in male groups. In addition, studies have shown that individuals who suffer from social exclusion are afraid of interpersonal communication, and individuals with high neuroticism tend to experience negative emotions more easily and be more sensitive to negative evaluations, which may lead to anxiety or depression [7]. Furthermore, the causes of FPE and FNE, and in those who experience both FNE and FPE, does FNE appear before FPE in time, or vice versa? Do these fears appear at the same time? The social experiences and personal traits that affect FPE and FNE are also worthy of further study. In conclusion, examining the differences in positive and negative evaluation fears among different groups not only helps in understanding the unique behaviors of these groups when facing social evaluations but also helps clarify the relationship between the two types of evaluation fears to determine the unique sources of each type of evaluation fear.

Finally, the development of therapies targeting different types of evaluation fears is needed. First of all, considering the behavioral and neural differences between FNE and FPE, some traditional methods may not be fully applicable for treating different types of social anxiety. For example, researchers have attempted to help patients overcome social anxiety by getting them to eliminate negative vocabulary through attention bias modification training, which is effective for individuals with high FNE, but individuals with high FPE still resist change during training [29]. However, in another study, in which subjects were exposed to positive face stimuli during attentional bias training, it was found that their levels of positive evaluative fear were significantly reduced [78]. It has also been found that for the initial speech task, FNE was the main construct associated with social anxiety, but for the second speech task, FPE was the main cause of increased anxiety regardless of feedback type [79]. It has been found that even though standard cognitive-behavioral treatment protocols help to reduce both FNE and FPE, the reduction in FPE is smaller than that of FNE [26, 80]. Anxiety and depression comorbidity is a common clinical psychological phenomenon; however, positive appraisal of fear is a core cognitive trait by which social anxiety can be distinguished from depression [9, 81], and we should pay attention to the positive appraisal of fear. Therefore, in the future, different cognitive training methods (such as cognitive restructuring) can be developed specifically for FPE to improve symptoms related to “positivity impediment.” In addition, researchers can further explore the relationship between FNE and FPE by incorporating the similarities and differences between positive and negative evaluations of fear to improve treatments. For example, targeting the common emotional response characteristics of both types and combining emotion regulation with cognitive training methods can be attempted to develop new treatment approaches. Furthermore, different therapeutic approaches can be taken for different cultural contexts. As mentioned earlier, FNE is higher than FPE in collectivist cultural contexts, whereas FPE is higher than FNE in individualist cultures [46], so although cognitive behavioral therapy for social anxiety is effective in both individualist and collectivist countries [82, 83], for evaluating different cultural contexts of fears, targeted programs can be adopted. Additionally, different treatment plans can be developed based on the neural mechanisms underlying FNE and FPE differences. For example, as individuals high in FNE are characterized by rejection sensitivity and always expect to be rejected by others when interacting with them, showing a high tendency towards interpersonal avoidance, brain regions such as the VLPFC and the striatum are activated and show a high degree of attentiveness to social cues of rejection [59, 60]. So, for individuals with high FNE, transcranial electrical stimulation targeting the ventrolateral prefrontal lobe can be explored to help improve social anxiety symptoms. In conclusion, understanding the similarities and differences between FNE and FPE can assist researchers in developing targeted treatment plans for social anxiety based on the characteristics of positive and negative evaluation fears.

## 7. Conclusion

In summary, findings from the systematic review extend the BFoEM and provide neural evidence that FPE and FNE are distinct yet correlated, trait-based social evaluative fears that uniquely contribute to social anxiety symptomatology. These findings integrate the evaluation of fear-related theories, internal neural mechanisms, and external expressions, which support the cognitive, emotional, and behavioral processes underlying social anxiety. Potential clinical implications highlight FPE and FNE as co-occurring cognitive features of social anxiety and provide insights into the high comorbidity of SAD and other mental disorders.

## Data Availability

This manuscript is a review article and does not involve generating original data. We have conducted a comprehensive review of the literature in the relevant fields. All findings are from published literature, and details of the sources can be found in the manuscript.
